# Bias-Free Optically Controlled Pattern-Reconfigurable Planar Antenna

**DOI:** 10.3390/s26144589

**Published:** 2026-07-20

**Authors:** Karam Younus, Khalil Sayidmarie, Kamel Sultan, Amin Abbosh

**Affiliations:** 1 School of Electrical Engineering and Computer Science, University of Queensland, Brisbane, QLD 4072, Australia; k.sultan@uq.edu.au (K.S.); a.abbosh@uq.edu.au (A.A.); 2 College of Electronics Engineering, Ninevah University, Mosul 41002, Iraq; kh.sayidmarie@uoninevah.edu.iq

**Keywords:** bias-free reconfiguration, light-dependent resistor (LDR), pattern-reconfigurable antennas, sub-6 GHz, Wi-Fi 6E

## Abstract

An antenna-level bias-line-free, optically controlled pattern-reconfigurable planar antenna for wideband sub-6 GHz applications is presented. The proposed antenna consists of four tapered-slot radiating units arranged in a compact planar configuration and excited through a reconfigurable feeding network incorporating four light-dependent resistors (LDRs). By selectively illuminating LDRs using aligned LEDs, the corresponding feed arms are activated without DC-bias lines embedded in the antenna itself, enabling pattern switching while avoiding a complicated biasing network. The antenna supports ten radiation states, including four principal directions (0°, 90°, 180°, and 270°), four diagonal directions (45°, 135°, 225°, and 315°), and two dual-beam excitations (0°/180° or 90°/270°). Measured results show impedance matching over 3.3–6.7 GHz for all states (at −10 dB |S_11_| reference), with a realized gain of 5.8–6.3 dBi and a total radiation efficiency of 86–90% across the operating band. The proposed approach offers a compact, wideband, and practically implementable platform for bias-free pattern reconfiguration suitable for coverage and interference adaptation in sub-6 GHz wireless systems.

## 1. Introduction

As modern wireless systems continue to evolve, antenna platforms are increasingly required to be compact, highly integrable, and multifunctional, enabling more capabilities within limited physical footprints [1,2,3,4]. In this context, reconfigurable antennas have emerged as an effective approach to combine multiple functionalities into a single platform, reducing the need for separate antennas and improving form-factor efficiency [5]. Depending on the controlled electromagnetic property, reconfigurable antennas are commonly categorized as frequency, polarization, or pattern-reconfigurable structures [6,7,8,9,10]. Compared with conventional fixed antennas, reconfigurable solutions provide enhanced operational flexibility, including improved interference management, adaptive link quality, and the ability to meet diverse deployment constraints across dynamic propagation environments [11].

**Figure 1:**
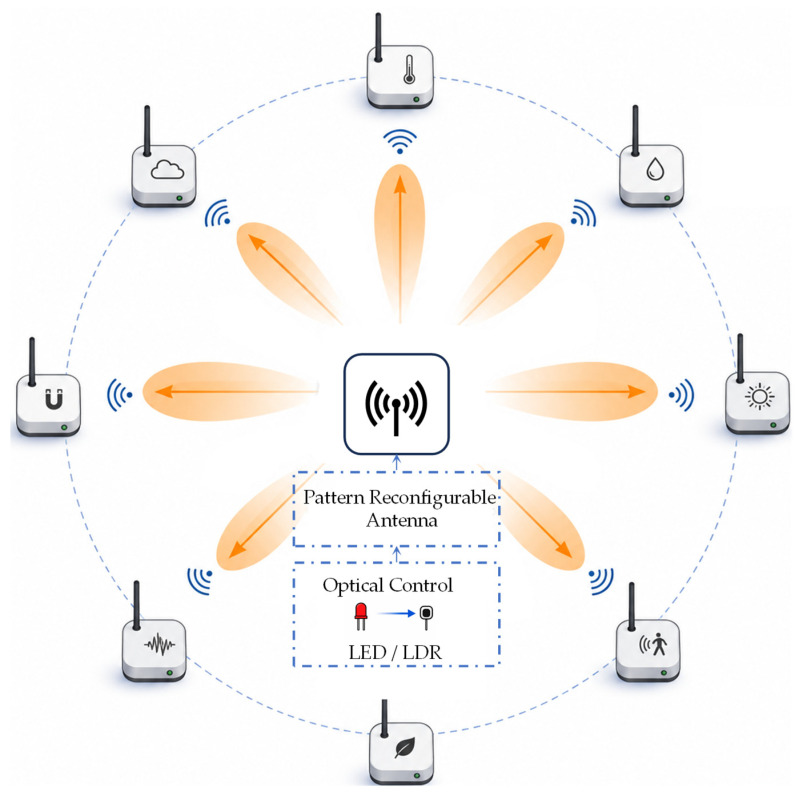
Concept illustration of the proposed optically controlled pattern-reconfigurable antenna as the wireless front-end of a smart sensor node/IoT system. The central antenna switches its radiation pattern through optical control of the LED/LDR pairs, enabling directional communication with surrounding sensor nodes or gateway devices.

Among these categories, pattern-reconfigurable antennas are particularly attractive for sub-6 GHz systems because they can modify the radiation direction, beam shape, or coverage region while maintaining the same operating band and polarization [2,12] (see Figure 1). Pattern reconfiguration is typically achieved by altering the current distribution on the radiator and/or ground through controllable perturbations in the radiating structure or feeding mechanism [11]. Common pattern-reconfigurable antenna realizations can be broadly grouped into: (i) integrating switching/tunable elements to modify surface currents or effective electrical lengths [3,13,14,15]; (ii) introducing parasitic structures (e.g., Yagi–Uda-inspired directors/reflectors) to reshape the beam [2,16,17,18,19]; and (iii) exciting multiple ports or modes to selectively activate different radiation states, including mode-combination approaches such as even–odd excitations [20,21].

To date, electrically controlled reconfiguration remains the most prevalent, typically relying on active devices such as PIN diodes, varactor diodes, and radio-frequency microelectromechanical-system (RF-MEMS) switches [22,23,24]. However, a persistent limitation of these approaches is the requirement for bias networks and associated control circuitry [25]. In practice, such bias networks are rarely RF-transparent: they can couple in RF currents, introduce lumped parasitics, and constrain the physical layout. They often require vias, RF chokes, DC-blocking capacitors, or isolation features, which may degrade impedance matching and radiation performance [20]. These effects become more pronounced as antenna dimensions shrink. At millimeter-wave frequencies, device packaging and bias routing can measurably perturb return loss and patterns unless carefully mitigated, and similar issues can arise at sub-6 GHz when bias traces share current paths or introduce ground discontinuities. Removing the bias network, therefore, addresses these compromises at their root [26,27,28].

Moreover, each common electrical switching technology introduces practical overheads. PIN diodes typically require additional RF/DC bias blocks and sufficient forward current to achieve a low-resistance ON state [29]. Varactor-based tuning enables continuous control with minimal reverse-bias current, but still depends on bias circuitry that adds loss and design complexity. RF-MEMS switches provide excellent linearity and near-zero static power, yet often require actuation voltages on the order of tens of volts and corresponding driver circuits, which increases integration burden even when average power is low [16,24]. Pressure-actuated liquid-metal switches have been reported for combined frequency tuning (2.28–2.5 GHz) and pattern switching in a Yagi configuration; however, switching relies on applied mechanical pressure [2]. Collectively, these device- and system-level trade-offs motivate bias-free reconfiguration strategies that decouple antenna reconfigurability from electrical biasing, which is particularly attractive for energy-constrained platforms [22,29,30,31].

Beyond eliminating conventional RF/DC bias lines, optical control enables a form of spatially driven antenna reconfiguration in which the radiation state can be selected according to the position or intensity of incident light. This capability is attractive for smart environments where existing lighting infrastructure, directed LEDs, or localized optical triggers can be used to align antenna coverage toward specific users, zones, or communications scenarios. For example, an IoT access point, indoor sensing node, or smart infrastructure unit could adapt its radiation pattern toward an illuminated region without requiring phase shifters, complex beamforming networks, or embedded electrical bias routing. This makes the concept suitable for adaptive coverage, interference mitigation, and context-aware wireless links in compact sub-6 GHz systems.

Optical-control-based antenna reconfiguration has also been demonstrated using photoconducting switches and other optically controlled elements. In [32], photoconducting switches were used to realize both frequency and beam reconfiguration. In [33], optical control was applied to a planar pattern-reconfigurable antenna, while an optically controlled antenna array based on E-shaped elements was reported in [34]. These works demonstrate the feasibility of optical antenna reconfiguration without direct DC bias lines on the radiating structure. However, they generally require specialized photoconductive materials, dedicated optical excitation, precise alignment, or additional optical-control hardware. In our previous work, a single LDR was embedded in a monopole antenna to optically change the effective current path and achieve bias-line-free frequency reconfiguration [31].

In contrast to the earlier LDR-based design [31], where illumination was used to modify the effective electrical length of a monopole radiator, the present work employs optically controlled LDRs within a reconfigurable feeding network to selectively activate different tapered-slot radiating units and generate multiple beam states. The proposed antenna provides practical coverage and interference adaptation over 3.3–6.7 GHz, covering sub-6 GHz 5G mid-band services and part of the 6-GHz Wi-Fi spectrum. Unlike conventional electrically biased switching networks, optical control using LDRs enables radiation-state selection without RF/DC bias lines, simplifying integration and mitigating bias-line coupling and pattern distortion. The proposed approach, therefore, provides a compact platform that demonstrates pattern switching with minimal biasing complexity while maintaining stable wideband performance.

From the sensors perspective, the proposed antenna is intended as a reconfigurable wireless front-end for smart sensor nodes and IoT monitoring systems, rather than as a sensing transducer itself. In practical smart-sensing deployments, the sensor-node orientation, gateway direction, surrounding objects, and multipath environment may change. Therefore, a fixed-pattern antenna may not always provide a stable wireless link. Optically controlled pattern switching enables the sensor node to select a suitable radiation direction toward the gateway or surrounding nodes, or to avoid an unfavorable propagation direction, improving link robustness without mechanical rotation (see Figure 1). Moreover, the LEDs can be driven by the sensor-node microcontroller, while the antenna RF structure remains free from antenna-level bias lines. This avoids additional biasing conductors on the antenna and reduces possible interaction with the RF current paths, such as impedance perturbation, parasitic radiation, and additional coupling paths. Figure 1 illustrates a representative smart-sensing/IoT scenario in which the proposed optically controlled pattern-reconfigurable antenna acts as the wireless front-end of a sensor node. The antenna switches its radiation pattern through LED/LDR optical control to support directional communication with surrounding sensor nodes or an IoT gateway.

The rest of this paper is organized as follows. Section 2 describes the antenna configuration, LDR-based optical switching mechanism, working principle, and equivalent-circuit analysis of the feeding network. Section 3 presents the simulated and measured results, including impedance matching, gain, efficiency, radiation patterns, polarization performance, and sensitivity analysis. Section 4 compares the proposed antenna with previously reported pattern-reconfigurable antennas. Finally, Section 5 concludes the paper.

## 2. Antenna Design

### 2.1. Antenna Configuration

**Figure 2:**
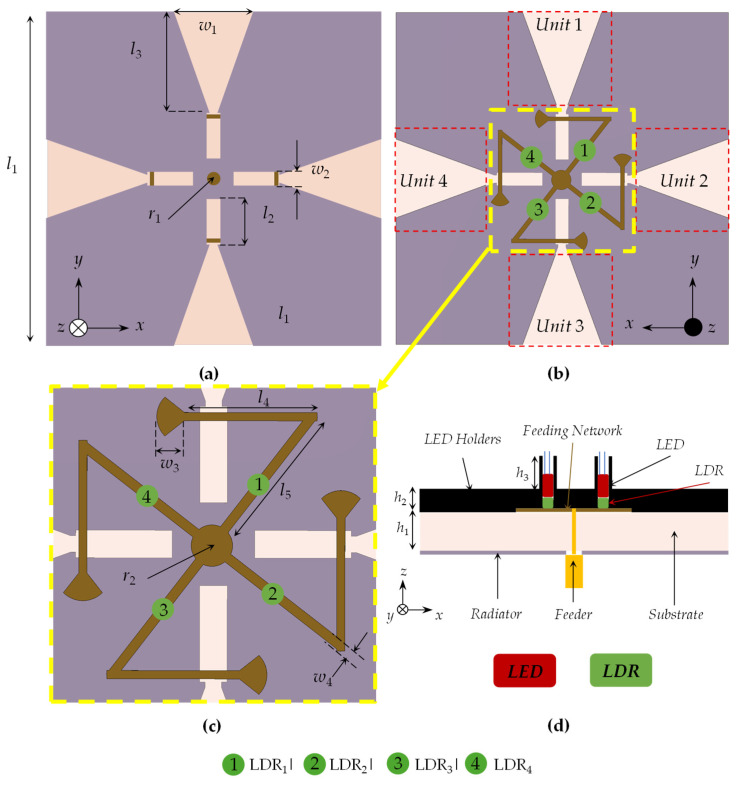
Geometry of the proposed antenna: (**a**) antenna top view, (**b**) antenna bottom view, (**c**) enlarged feeding network, and (**d**) schematic of the antenna side view. Dimensions in (mm): *l*_1_ = 50, *l*_2_ = 7, *l*_3_ = 14.6, *l*_4_ = 9.6, *l*_5_ = 12.1, *w*_1_ = 11.8, *w*_2_ = 2, *w*_3_ = 2, *w*_4_ = 0.6, *h*_1_ = 0.508, *h*_2_ = 0.35 and *h*_3_ = 10.

The geometry of the proposed pattern-reconfigurable antenna is illustrated in Figure 2. It comprises four reconfigurable radiating units, each implemented as a tapered-slot antenna and labeled Unit 1–Unit 4, which collectively form the planar radiator, together with a reconfigurable feeding network implemented using four curved microstrip lines terminated with fan-shaped baluns. The radiating units are excited through a microstrip-to-slot coupling transition, while the fan-shaped baluns are employed to improve impedance matching. Pattern reconfigurability is achieved by embedding four LDRs (LDR1–LDR4) within the feeding network, enabling optical switching between distinct states (ON/OFF). The antenna is fed by a 50 Ω coaxial port. The antenna geometry, key dimensions, feeding network, and LED/LDR arrangement are summarized in Figure 2a–d. The design is implemented on a Rogers RT-Duroid 5880 substrate with a thickness of 0.508 mm, for the antenna substrate and central feeding structure, with relative permittivity εr = 2.2 and loss tangent tanδ = 0.0009.

The LDR used in this design is the NSL-6112, a CdS-based photoresistor with a ceramic two-lead package, offering reliable visible-light sensitivity in the 400–700 nm range. The LDR resistance varies significantly with incident illumination. Under low-to-moderate illumination levels of 21–324 lux, the resistance is approximately 1.1–2 kΩ, whereas under near-dark conditions of ≤5 lux, the resistance can exceed 1.3 MΩ, effectively behaving as an open circuit [35]. Under stronger illumination (e.g., 1076 lux), the resistance decreases to approximately 170 Ω and can be further reduced to below 25 Ω at about 2000 lux, providing a low-resistance conductive path between the feeding lines [36]. In this design, each LDR is illuminated by an LED, and the incident illumination level (lux) is controlled via the LED drive (set by the applied drive voltage and corresponding current), enabling repeatable switching between the high- and low-resistance states.

The RF behavior of the LDRs was represented using an illumination-dependent resistance together with package parasitics. The values $R_{ON}=25\;\Omega$ and $R_{OFF}=1.3\;M\Omega$ correspond to illumination-dependent resistance states obtained from datasheet/characterization conditions and are used as the resistive part of the lumped RF equivalent model. At microwave frequencies, the effective impedance is modified by the LDR package and mounting parasitics. The RF equivalent circuit of the LDR is represented by an illumination-dependent resistance $R_{LDR}$, in parallel with a parasitic capacitance $C_{p}$, together with a series parasitic inductance $L_{p}$. The capacitance $C_{p}$ accounts for the parasitic coupling across the LDR terminals and mounting pads, while $L_{p}$ represents the metallic leads, solder pads, package, and physical mounting between the RF traces. The equivalent impedance is given by

(1) $$Z_{LDR}\left(f\right)=j2\pi fL_{p}+\left(R_{LDR}∥\frac{1}{j2\pi fC_{p}}\right).$$

Representative values of $L_{p}=0.4\;\mathrm{nH}$ and $C_{p}=0.022\;pF$ were used to estimate the RF impedance. These values are within the typical order used for compact RF component and microwave PIN-diode package models [37,38]. Using these values, the estimated $|Z_{ON}|$ values are 26.0, 27.8, and 30.2 Ω at 3, 5, and 7 GHz, respectively. The corresponding $|Z_{OFF}|$ values are 2403.9, 1434.3, and 1015.9 Ω, respectively. These estimates show that the LDR is not modeled as an ideal short or open circuit at microwave frequencies. In the ON state, the impedance is mainly governed by the illuminated resistance and the series inductive parasitic. In the OFF state, the high resistance is accompanied by parasitic capacitance, which may allow weak residual RF leakage at higher frequencies. Therefore, the practical RF behavior of the LDR-loaded structure is validated at the antenna level through the measured $|S_{11}|$ responses and radiation-pattern reconfiguration results.

The optical activation setup uses four red through-hole LEDs (Dialight 5219247F), each aligned with one LDR inside an individual black 3D-printed PLA optical channel. The LED has a peak wavelength of 635 nm and a luminous intensity of 25–60 mcd at a forward current of 10 mA [39]. In the prototype, each LED was driven from a 5 V supply through a 220 Ω series resistor. Considering the LED forward-voltage range of 2.2–3.0 V [39], the estimated LED current is 9.1–12.7 mA, which is below the maximum continuous forward current of 30 mA. The LED was mounted directly above the LDR surface with a clearance not exceeding 0.1 mm. Based on the LED luminous intensity, the expected illuminance is approximately 1000–2400 lux at a 5 mm distance; therefore, the near-contact LED/LDR arrangement provides sufficient illumination to drive the LDR into its low-resistance ON state. The short LED-to-LDR separation also minimizes optical attenuation and diffraction, supporting reliable switching. Possible light leakage is strongly suppressed by the black PLA cover, which separates the four LED/LDR pairs using individual optical channels and blocks ambient light. As a result, the illumination is confined mainly to the intended LDR, while adjacent LDRs remain in their high-resistance OFF states. Furthermore, the large resistance difference between the OFF state (>1 MΩ) and ON state (<25 Ω) provides a clear switching margin and improves the repeatability of the optical switching mechanism.

Regarding long-term reliability, neither the manufacturer documentation nor the reviewed literature specifies a quantitative lifetime in hours or switching cycles for the NSL-6112 LDR; therefore, such a value is not claimed here. According to the NSL-6112 datasheet, the LDR operates over −60 °C to +75 °C, with a maximum voltage rating of 100 V and a power dissipation rating of 50 mW [35]. For the proposed optical switching mechanism, long-term reliability is expected to be mainly governed by LED aging and gradual photocell resistance drift rather than mechanical wear. Reported LED lifetimes can range from 10,000 to 200,000 h depending on the operating conditions and application, while photocell resistance may drift by approximately 10% per year under continuous illumination and voltage bias [36]. Therefore, stable optical alignment, controlled LED driving, and limiting unnecessary continuous illumination are important for practical deployment. In the proposed design, each LED/LDR pair is fixed inside a black 3D-printed PLA holder, which maintains alignment, blocks ambient light, and reduces leakage to adjacent LDRs. Accelerated aging or switching-cycle tests were not performed in this work; therefore, the proposed method is not claimed to have a quantified lifetime or a longer lifetime than PIN-diode switching. Its main advantage is the elimination of antenna-level RF/DC bias lines while maintaining contactless optical control.

The term “bias-free” in this work refers to the antenna RF structure, where no DC bias lines, RF chokes, or biasing networks are connected to the radiating elements or feeding network. The LDRs are controlled optically rather than electrically through the antenna metallization. The LEDs are externally driven as part of the optical control setup; therefore, the proposed design is bias-line-free at the antenna level, while still requiring electrical power for LED illumination. This configuration avoids additional biasing conductors on the antenna and reduces unwanted interaction between the biasing network and the RF current paths.

### 2.2. Working Principle

The proposed antenna employs an annular feeding line with four branches to provide nominally balanced excitation among the four paths. The operating concept of the proposed antenna is governed by optical activation of the feed paths using four LDRs embedded in the feeding network. Each LDR is placed along one arm of the feeding structure and operates as an optically controlled ON/OFF switch. In the OFF state, the corresponding feed arm is disconnected, and the associated radiating unit is not excited. In the ON state, the LDR provides a conductive path that activates the corresponding feed arm and energizes the associated radiating unit. The beam direction is consequently determined mainly by the physical location of the active radiating unit(s).

Since the active units are excited with nominally equal phase through the symmetric feed, the beam direction for simultaneous activation of multiple units can be approximated by the vector sum of the active unit directions:

(2) $$\varphi_{beam}=\frac{\varphi_{i}+\varphi_{j}}{2}$$

where i and j denote the two adjacent active radiating units, and $\varphi_{i}$ and $\varphi_{j}$ are their nominal beam directions. Therefore, adjacent-unit excitation gives beam directions of 45°, 135°, 225°, and 315°, while opposite-unit excitation gives the dual-beam states along 0°/180° or 90°/270°, rather than a single tilted beam.

In the dark/low-illumination condition, the LDR exhibits very high resistance and behaves as an open circuit. Consequently, the associated feed arm is effectively disabled, preventing power delivery to that radiating unit. When a light source is applied to an LDR, its resistance drops, providing a conductive path that turns the corresponding feed arm ON. This enables RF power to reach the associated radiator, which becomes active and contributes to the overall radiation.

With four LDRs (LDR_1_–LDR_4_), the antenna supports multiple controllable excitation combinations. By illuminating a single LDR at a time, four primary radiation states are obtained, corresponding to beams directed nominally toward 0°, 90°, 180°, and 270°, respectively. Additional intermediate states can be realized by illuminating two adjacent LDRs simultaneously, which activates two neighboring radiating units and produces diagonal beams toward approximately 45°, 135°, 225°, and 315°. Moreover, illuminating two opposite LDRs enables dual-unit excitation along the principal axes, generating pattern states associated with 0°/180° or 90°/270° excitations, depending on the selected pair. All switching states are listed in Table 1.

**Figure 3:**
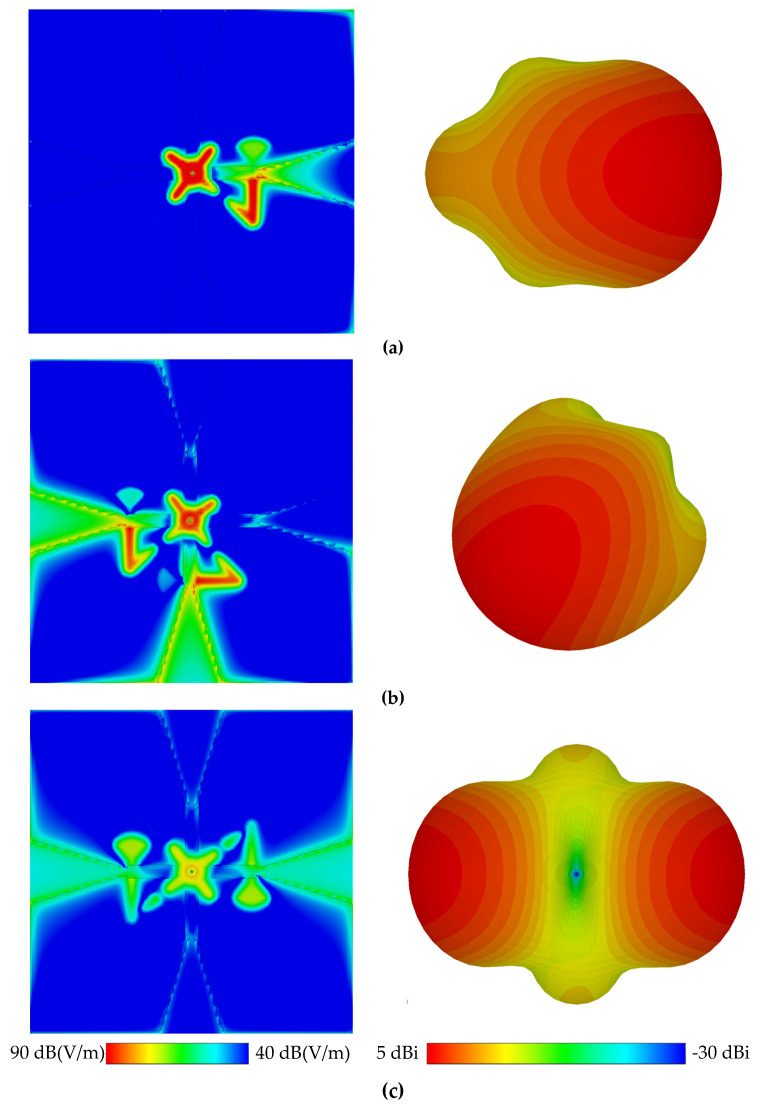
Simulated electric-field distributions (left) and the corresponding 3-D radiation patterns (right) examples at 5 GHz for (**a**) State 1, (**b**) State 5, and (**c**) State 10.

The electric-field distributions shown in Figure 3 illustrate how the individual, orthogonal, and symmetric radiating elements operate. They also indicate the ON/OFF conditions of the LDRs. When an LDR is in the OFF state, the corresponding antenna unit is effectively suppressed. Conversely, when the LDR is ON, that unit radiates efficiently, enabling the formation of various combined radiation patterns.

Through these optically controlled switching combinations, the proposed design provides flexible pattern reconfiguration without DC-bias lines, while maintaining a compact planar implementation.

### 2.3. Equivalent-Circuit Analysis of the Feeding Network

The proposed coaxial feed excites four microstrip branches that are connected to the four radiating units through series LDR elements. When the illumination state changes, the effective impedance of one (or two) branches varies due to the LDR resistance and the corresponding radiator loading. Nevertheless, the simulated |S_11_| remains relatively stable across the ten switching states. An explanation can be obtained by representing the input as the parallel combination of the effective branch impedances:

(3) $$Z_{in}=Z_{1}||Z_{2}||Z_{3}||Z_{4}.$$

To examine the sensitivity of *Z_in_* to a change in one branch, consider *Z*_2_ = *Z*_3_ = *Z*_4_ = *Z*. The parallel combination of the unchanged three branches is *Z*/3, and thus

(4) $$Z_{in}=\frac{Z_{1}(Z/3)}{\left(Z_{1}+(Z/3)\right)}=\frac{Z_{1}\;Z}{3Z_{1}+Z}.$$

Differentiating with respect to *Z*_1_ yields

(5) $$\frac{dZ_{in}}{dZ_{1}}=\frac{Z^{2}}{{\left(3Z_{1}+Z\right)}^{2}}.$$

For the nominal symmetric case where *Z*_1_ ≈ *Z*, the sensitivity reduces to

(6) $$\frac{dZ_{in}}{dZ_{1}}=\frac{1}{16},$$

indicating that a perturbation in a single branch produces a substantially attenuated change at the input. This parallel-loading property provides a useful qualitative rationale for the observed robustness of the input matching across different switching states. This simplified model serves as an intuitive explanation. However, full-wave simulations capture the complete frequency-dependent behavior of the coupled structure and radiators.

## 3. Results and Discussion

**Figure 4:**
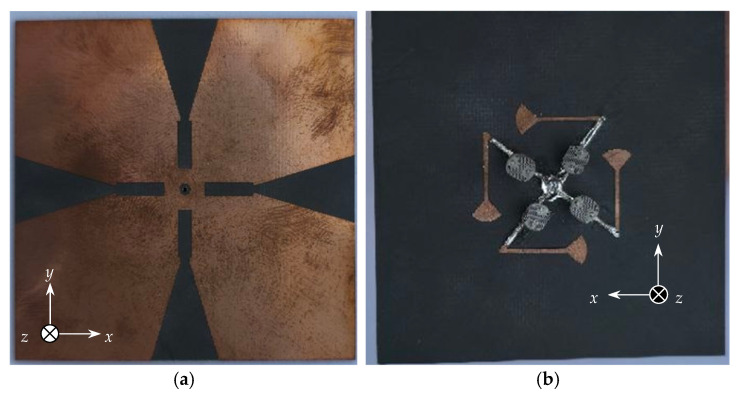

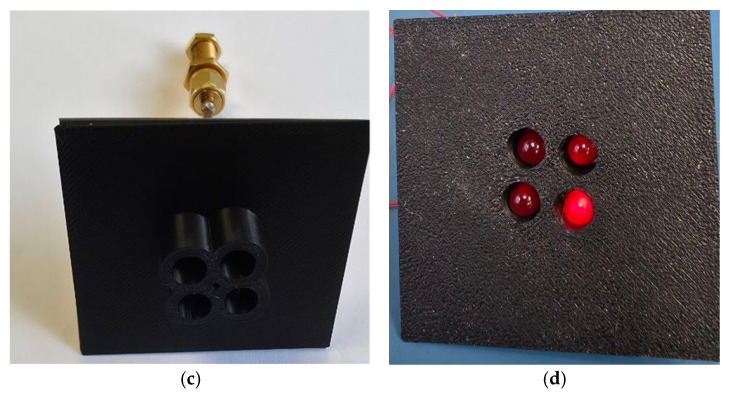
Fabricated antenna prototype and optical-control setup. (**a**) Front view. (**b**) Back view. (**c**) Antenna integrated with the LED holder. (**d**) Example of one reconfiguration state with a single LED turned ON.

The proposed antenna was simulated and experimentally validated to evaluate its impedance matching, radiation performance, and pattern-reconfiguration capability. The fabricated prototype and optical-control setup are shown in Figure 4.

The 3D-printed LED-holder cover, shown in Figure 2d and Figure 4c, is an essential part of the optical switching system because it holds the LEDs in fixed alignment with the LDRs and reduces unwanted ambient-light effects. The cover was included in the full-wave simulation model, and the feeding-network dimensions were optimized with the cover present. Therefore, the simulated and measured results reported below already include its effect. Since the cover is placed over the feeding-network region and does not cover the tapered-slot radiating apertures, it does not obstruct the main radiating paths of the active units.

**Figure 5:**
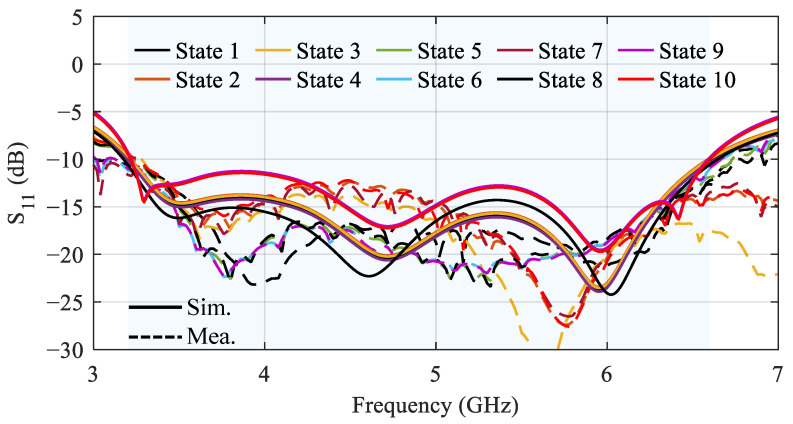
Simulated and measured |S_11_| of the proposed antenna for ten pattern-reconfigurable states.

The simulated and measured $|S_{11}|$ responses for States 1–10 are presented in Figure 5. Across all switching states, the antenna maintains good impedance matching, with a common $|S_{11}|$ bandwidth of 3.2–6.6 GHz in simulation and 3.3–6.7 GHz in measurement. This confirms that the wide operating band is preserved under the ten pattern reconfigurations. The small deviations between the simulated and measured $|S_{11}|$, mainly in the response depths at resonance, are attributed to practical effects such as soldering pads and joints, the coaxial connector transition, and fabrication tolerances. Nevertheless, all measured states maintain $|S_{11}|\;<\;-10$ dB over the intended operating band, confirming that wideband impedance matching is preserved.

**Figure 6:**
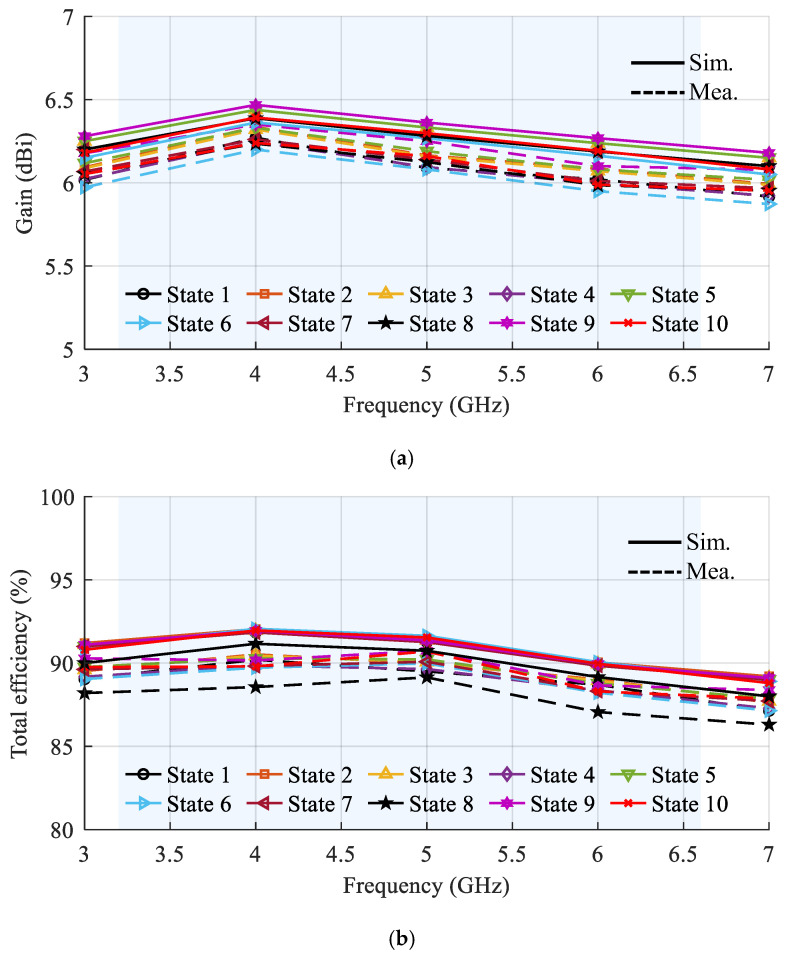
Simulated and measured antenna performance across the operating band: (**a**) realized gain and (**b**) total efficiency.

Across the operating band, the realized gain exhibits stable performance, varying from 6.2 to 6.4 dBi in simulation and from 5.8 to 6.3 dBi in measurement, as shown in Figure 6a. The simulated and measured total radiation efficiencies are summarized in Figure 6b, where high efficiencies are achieved, ranging from 88% to 92% in simulation and from 86% to 90% in measurement. These results indicate that the proposed optical switching approach preserves stable radiation performance across the reconfigurable states.

**Figure 7:**
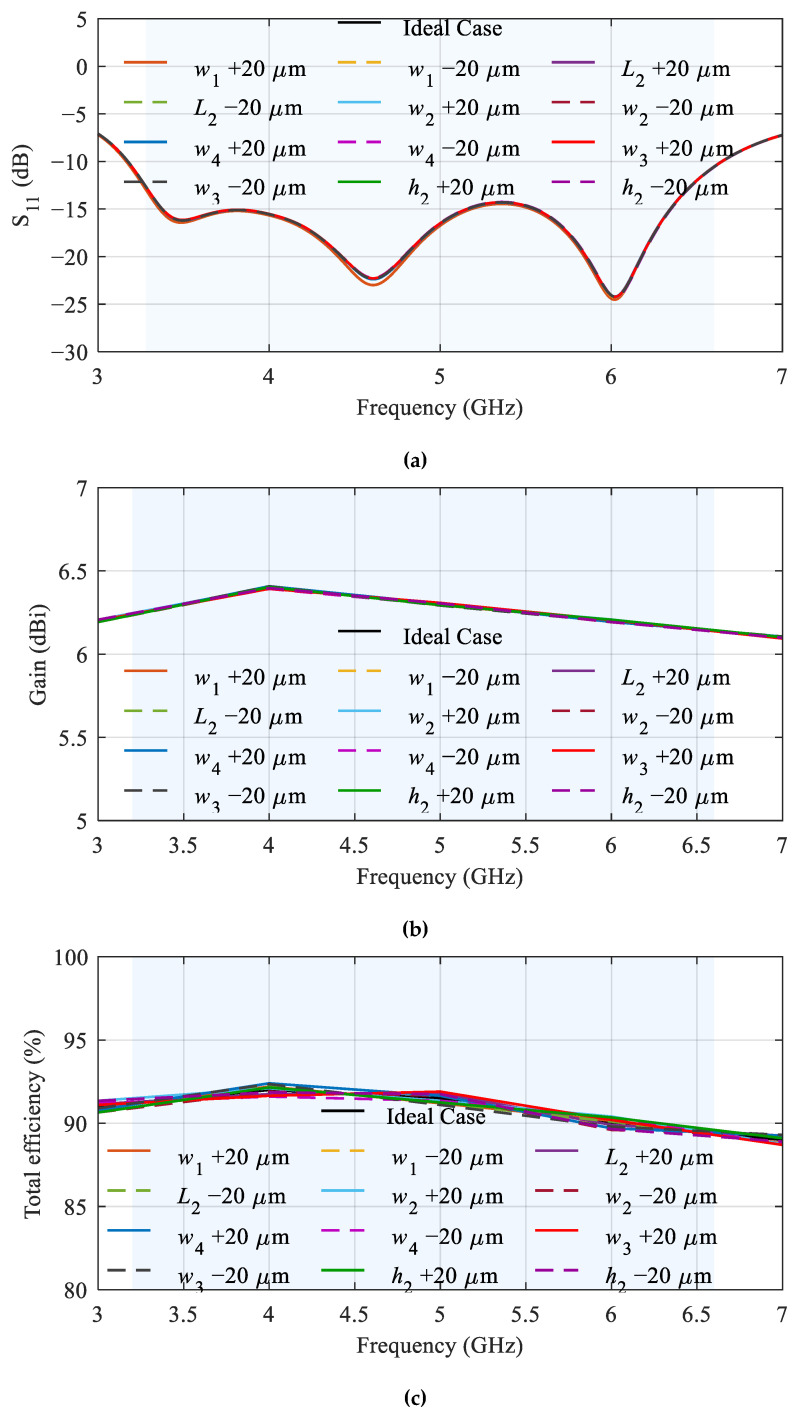
Sensitivity analysis of the proposed antenna under ±20 μm variations in the key geometrical parameters $w_{1}$, $w_{2}$,$\;w_{3},\;w_{4}$, $l_{2}$ and $h_{2}$: (**a**) reflection coefficient, (**b**) realized gain, and (**c**) radiation efficiency.

To evaluate the robustness of the proposed design against fabrication tolerances, a sensitivity analysis was performed by varying the key geometrical parameters $w_{1}$, $w_{2}$, $w_{3}$, $w_{4}$, $l_{2}$, and $h_{2}$ by ±20 μm [40]. These parameters were selected because they mainly affect the tapered-slot radiator, feed transition, and feeding-network impedance. Each parameter was varied individually while the others were kept at their nominal values. Figure 7a shows the corresponding $|S_{11}|$ responses, while Figure 7b and Figure 7c show the realized gain and radiation efficiency for State 1, respectively. This state was selected as a representative single-arm excitation case because the other principal single-arm states are rotationally symmetric. The results confirm that the impedance bandwidth, gain, and efficiency remain stable under the considered tolerances.

**Figure 8:**
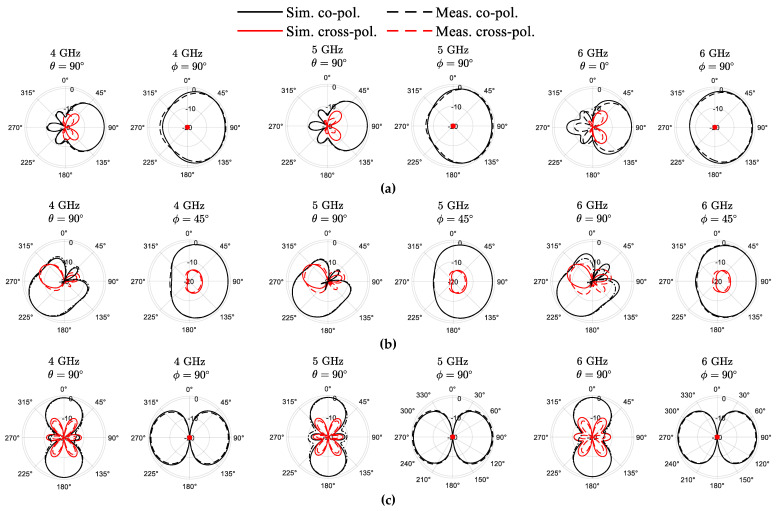
Simulated and measured normalized co-polarized and cross-polarized radiation patterns at 4, 5, and 6 GHz for representative switching states: (**a**) State 1, (**b**) State 5, and (**c**) State 10. For each state, the azimuth-plane cut (*θ* = 90°) and the corresponding principal elevation-plane cut are shown.

To evaluate the radiation stability and polarization performance, two-dimensional co-polarized and cross-polarized radiation patterns are presented in Figure 8 for three representative switching states. State 1 is selected as a principal single-beam case, State 5 represents a diagonal single-beam case, and State 10 represents a dual-beam excitation case. The patterns are plotted at 4, 5, and 6 GHz, corresponding to the lower, middle, and upper parts of the operating band. For each state, the azimuth-plane cut *θ* = 90° and the corresponding principal elevation-plane cut are shown. The results indicate that the co-polarized component remains dominant in the intended radiation direction, while the cross-polarized component remains lower. The main radiation behavior is also preserved across the three frequencies, confirming stable wideband pattern performance.

**Figure 9:**
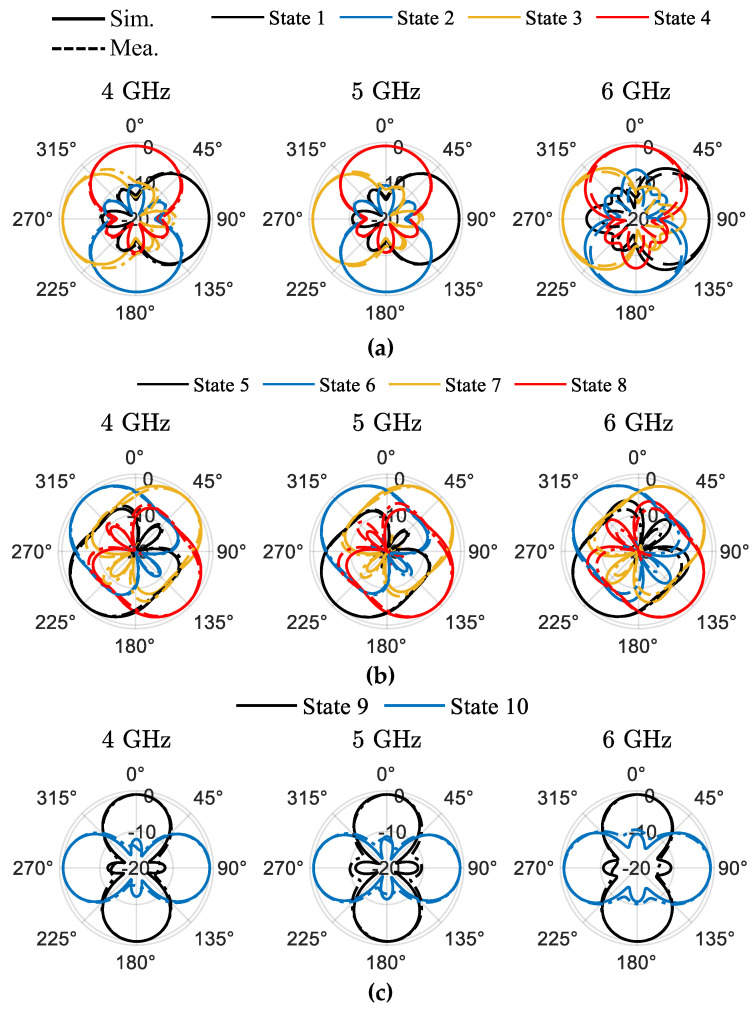
Simulated and measured normalized realized gain patterns in the azimuth plane *θ* = 90°, showing the radiation coverage of the proposed antenna for: (**a**) States 1–4, (**b**) States 5–8, and (**c**) States 9 and 10.

To further illustrate the full pattern-reconfiguration capability, the azimuth-plane radiation patterns *θ* = 90° for all ten switching states are shown in Figure 9. States 1–4 steer the main beam toward 0°, 90°, 180°, and 270°, respectively; States 5–8 generate diagonal beams toward 45°, 135°, 225°, and 315°; and States 9–10 correspond to dual-unit excitations along the principal axes, producing patterns associated with 0°/180° and 90°/270° excitations, respectively. These results confirm that the proposed antenna provides the intended principal, diagonal, and dual-beam radiation states.

To provide state-by-state quantitative validation, Table 2 summarizes the measured beam direction, pointing error, HPBW, and peak realized gain at 5 GHz for all ten switching states.

## 4. Comparison

The proposed LDR-based pattern-reconfigurable antenna demonstrates a favorable tradeoff among bandwidth, electrical size, number of states, and peak gain compared with previously reported designs. As summarized in Table 3, this work achieves a measured operating band of 3.3–6.7 GHz, corresponding to a fractional bandwidth of 68.0%, while providing ten radiation states using only four optically controlled LDRs. Several reported antennas in [2,3,7,41,42,43] exhibit narrower fractional bandwidths of 1.0–11.1%, despite offering multiple switching states.

Other designs, such as [15,44,45], provide moderate bandwidths of 8.0–33.6%, but with fewer states or larger profiles. Although [46] reports a slightly wider bandwidth of 71.3% and a marginally higher peak gain of 6.4 dBi, it requires a substantially larger electrical footprint of 1.57 × 1.57 *λ_l_*. Similarly, ref. [41] achieves a higher peak gain of 7.3 dBi but with a much narrower bandwidth of 2.4%. Compared with these designs, the proposed antenna combines wide bandwidth, ten radiation states, compact RF-board size, and competitive gain, while using only four optical switching elements. Therefore, it provides a balanced wideband solution with simplified antenna-level bias-line-free optical control for pattern-reconfigurable platforms.

## 5. Conclusions

An antenna-level bias-line-free, optically controlled pattern-reconfigurable planar antenna has been presented for wideband sub-7 GHz operation. Pattern switching is achieved by integrating four LDRs into a reconfigurable feed network that selectively excites four tapered-slot radiating units under LED illumination, thereby eliminating antenna-level DC bias lines and mitigating bias-network coupling and parasitic effects. The antenna provides ten reconfigurable radiation states, enabling beam switching toward the four principal and four diagonal azimuth directions, in addition to two dual-beam excitations, which support flexible coverage shaping and interference-aware operation. Across all states, the antenna maintains a common measured wide impedance bandwidth of 3.3–6.7 GHz, while preserving stable performance with a realized gain of 5.8–6.3 dBi and a total efficiency of 86–90%. These results confirm that optical control using LDRs can realize practical wideband pattern reconfiguration with simplified integration and competitive radiation performance for sub-6 GHz platforms.

## Figures and Tables

**Table 1:** LDR switching states and corresponding beam directions.

States	LDR1	LDR2	LDR3	LDR4	Direction
State 1	ON	OFF	OFF	OFF	0°
State 2	OFF	ON	OFF	OFF	90°
State 3	OFF	OFF	ON	OFF	180°
State 4	OFF	OFF	OFF	ON	270°
State 5	ON	ON	OFF	OFF	45°
State 6	OFF	ON	ON	OFF	135°
State 7	OFF	OFF	ON	ON	225°
State 8	ON	OFF	OFF	ON	315°
State 9	ON	OFF	ON	OFF	0° + 180°
State 10	OFF	ON	OFF	ON	90° + 270°

**Table 2:** Quantitative radiation-pattern parameters for the ten switching states at 5 GHz.

State	Nominal Direction	Measured Beam Direction	Pointing Error	HPBW (Sim./Meas.)	Peak Realized Gain (dBi) (Sim./Meas.)
State 1	0°	2°	+2°	88.6°/88.4°	6.28/6.08
State 2	90°	92°	+2°	88.7°/88.7°	6.29/6.16
State 3	180°	182°	+2°	88.7°/88.8°	6.33/6.16
State 4	270°	272°	+2°	88.6°/88.7°	6.36/6.13
State 5	45°	45°	0°	82.7°/82.7°	6.33/6.18
State 6	135°	136°	+1°	82.7°/82.7°	6.26/6.08
State 7	225°	226°	+1°	82.6°/82.7°	6.28/6.13
State 8	315°	316°	+1°	82.5°/82.5°	6.28/6.12
State 9	0°/180°	0°/180°	0°	58.2°/58.1°	6.36/6.24
State 10	90°/270°	90°/270°	0°	58.3°/58.2°	6.29/6.16

Note: For States 9 and 10, the listed HPBW corresponds to the main lobes of the dual-beam pattern, which have nearly identical beamwidths.

**Table 3:** Comparison with previous pattern reconfigurable antennas.

Ref.	RF-Board Size (*λ_l_*)	Bandwidth GHz, FBW	State/Switches	Peak Gain dBi
[1]	0.72 × 0.70 × 0.009	2.6–3.5, (29.51%)	4/8	2.6
[2]	0.53 × 0.490 × 0.006	2.28–2.5, (9.2%)	4/4	6.0
[3]	2.4 × 2.4 × 0.03	4.65–5.2, (11.1%)	5/5	9.3
[7]	0.7 × 0.7 × 0.0106	4.17–4.5, (7.6%)	10/8	4.2
[15]	0.56 × 0.56 × 0.0057	2.25–3.16, (33.6%)	4/4	5.0
[44]	0.88 × 0.80 × 0.017	8.8–11.2, (24%)	8/8	5.2
[45]	0.46 × 0.46 × 0.1022	3.4–3.7, (8.0%)	8/4	4.9
[41]	0.81 × 0.77 × 0.0242	2.42–2.48, (2.4%)	9/4	7.3
[42]	0.41 × 0.41 × 0.0138	2.065–2.085, (1.0%)	4/0	3.0
[43]	0.31 × 0.31 × 0.0025	1.505–1.525, (1.3%)	3/2	5.4
[46]	1.57 × 1.57 × 0.0079	1.57–3.31, (71.3%)	4/4	6.4
This work	0.55 × 0.55 × 0.0056	3.3–6.7, (68.0%)	10/4 (LDR)	6.3

## Data Availability

The original contributions presented in this study are included in the article. Further inquiries can be directed to the corresponding author.
